# Comparative Analysis of Proteome-Wide Lysine Acetylation in Juvenile and Adult *Schistosoma japonicum*

**DOI:** 10.3389/fmicb.2017.02248

**Published:** 2017-11-21

**Authors:** Qing Li, Nan Zhao, Mu Liu, Haimo Shen, Lin Huang, Xiaojin Mo, Bin Xu, Xumin Zhang, Wei Hu

**Affiliations:** ^1^State Key Laboratory of Genetic Engineering, Ministry of Education Key Laboratory of Contemporary Anthropology, Collaborative Innovation Center for Genetics and Development, School of Life Sciences, Fudan University, Shanghai, China; ^2^National Institute of Parasitic Diseases, Chinese Center for Disease Control and Prevention, WHO Collaborating Centre for Tropical Diseases, National Center for International Research on Tropical Diseases, Ministry of Science and Technology, Key Laboratory of Parasite and Vector Biology, Ministry of Health, Shanghai, China

**Keywords:** *Schistosoma japonicum*, lysine acetylation, proteome, development, post translation modification

## Abstract

Schistosomiasis is a devastating parasitic disease caused by tremotodes of the genus *Schistosoma*. Eggs produced by sexually mature schistosomes are the causative agents of for pathogenesis and transmission. Elucidating the molecular mechanism of schistosome development and sexual maturation would facilitate the prevention and control of schistosomiasis. Acetylation of lysine is a dynamic and reversible post-translational modification playing keys role in many biological processes including development in both eukaryotes and prokaryotes. To investigate the impacts of lysine acetylation on *Schistosoma japonicum* (*S. japonicum*) development and sexual maturation, we used immunoaffinity-based acetyllysine peptide enrichment combined with mass spectrometry (MS), to perform the first comparative analysis of proteome-wide lysine acetylation in both female and male, juvenile (18 days post infection, 18 dpi) and adult (28 dpi) schistosome samples. In total, we identified 874 unique acetylated sites in 494 acetylated proteins. The four samples shared 47 acetylated sites and 46 proteins. More acetylated sites and proteins shared by both females and males were identified in 28 dpi adults (189 and 143, respectively) than in 18 dpi schistosomula (76 and 59, respectively). More stage-unique acetylated sites and proteins were also identified in 28 dpi adults (494 and 210, respectively) than in 18 dpi schistosomula (73 and 44, respectively). Functional annotation showed that in different developmental stages and genders, a number of proteins involving in muscle movement, glycometabolism, lipid metabolism, energy metabolism, environmental stress resistance, antioxidation, etc., displayed distinct acetylation profiles, which was in accordance with the changes of their biological functions during schistosome development, suggesting that lysine acetylation modification exerted important regulatory roles in schistosome development. Taken together, our data provided the first comparative global survey of lysine acetylation in juvenile and adult *S. japonicum*, which would deepen our understanding of the molecular mechanism of schistosome development and sexual maturation, and provide clues for the development of new anti-schistosome strategies.

## Introduction

Schistosomiasis is one of the most prevalent tropical diseases and remains a major public health problem with approximately 200 million people infected worldwide and around 779 million at the risk of infection (Wang et al., 2008; Cao et al., 2016). Schistosomiasis is a disease caused by the parasitic trematodes of the genus *Schistosoma*. Schistosomiasis *japonica* mainly distributed in East Asia, especially in China and the Philippines. Currently, no vaccine is available for schistosomiasis. Praziquantel (PZQ) is the only effective drug available for treating schistosomiasis, but only effective against adult schistosomes and less effective against juvenile worms. Extensive utilization of PZQ increases the risk of drug resistance (Gonnert and Andrews, 1977; Doenhoff and Pica-Mattoccia, 2006). Therefore, development of new anti-schistosome strategies is critical for the control of schistosomiasis.

Schistosomes have a complex life cycle including seven developmental stages with distinctly different morphologies. After being released from the snails, the free-swimming cercariae infect mammalian hosts by penetrating through their skin, then transform into schistosomula, the juvenile form of the parasite. The schistosomula migrate via the circulatory system to the lungs of the definitive host, and then move to the liver to pair. As pairs they arrive at mesenteric veins to reach their sexual maturation. Stimulations from the males lead to growth and reproductive system development of the females (Armstrong, 1965; Erasmus, 1973; LoVerde and Chen, 1991). The mature females produce a large number of eggs which are the source for transmission, as well as the causative agents for pathogenesis when they trigger immune-mediated granuloma in host liver. Therefore, understanding how female sexual maturation is regulated would provide us key molecular information and clues for the development of new anti-schistosome strategies.

Post-translational modifications (PTM) of proteins are key biological processes that regulate protein functions, such as protein activity, protein stability and protein-protein interactions by adding functional groups to amino acids to change their chemical structures (Mann and Jensen, 2003). As one of the most significant covalent modification, protein lysine acetylation is a reversible and dynamic modification catalyzed by lysine acetyltransferases (KATs), a diverse family of enzymes that utilize acetyl-CoA as acetyl group donor. The primary roles lysine acetylation plays in histone proteins and transcription-associated proteins in nucleus have been extensively studied since 50 years ago (Phillips, 1963; Allfrey et al., 1964). As a widespread PTM that may rival protein phosphorylation (Kouzarides, 2000), lysine acetylation also has prominent impacts on mRNA splicing, mRNA transport, translation, protein activity, protein stability, protein interactions, cytoskeleton dynamics, and cellular metabolism (Spange et al., 2009; Close et al., 2010). Recently, with the progress in mass spectrometry (MS)-based proteomics and antibody-based affinity enrichment, high throughput identification of lysine-acetylated sites and proteins has been widely investigated. Proteome-wide lysine acetylation has been extensively studied in plant (Fang et al., 2015), bacteria (Zhang et al., 2009; Xie et al., 2015), yeast (Henriksen, 2012), *Drosophila melanogaster* (Weinert et al., 2011), human cells (Kim et al., 2006; Choudhary et al., 2009; Zhao et al., 2010), as well as the protozoan parasites *Toxoplasma gondii* and *Plasmodium falciparum* (Jeffers and Sullivan, 2012; Miao et al., 2013). The first study of lysine acetylation in *Schistosoma japonicum* was also reported, in which 2393 acetylated sites in 1109 acetylated proteins were identified from 42 dpi adult worms (Hong et al., 2016). We believe comparative studies of acetylation profiles in different developmental stages will identify differentially acetylated proteins, and by investigating how acetylation of these proteins affects their biological functions, the impacts of protein lysine acetylation on *S. japonicum* developmental regulation will be further elucidated. However, such studies have not been reported to date.

In this study, 18 dpi juvenile and 28 dpi adult *S. japonicum*, which represented the two stages before and after sexual maturation, were collected as samples to investigate their scopes of acetylation using the pan anti-acetyllysine antibody enrichment and the highly sensitive LC-ESI-MS/MS-based proteomics. We identified 874 unique acetylated sites in 494 acetylated proteins in total, of which 46 acetylated proteins were shared by all samples. Our analysis suggested that acetylation participated in the regulation of development and sexual maturation of *S. japonicum*. To our knowledge, this is the first comparative acetylomic study between juvenile and adult *S. japonicum*. Our study deepened our understanding of the impacts of protein lysine acetylation on biological functions during the sexual maturation of *S. japonicum*.

## Materials and methods

### Ethics statement

All animal experiments were conducted according to the Guidelines for the Care and Use of Laboratory Animals of the Ministry of Science and Technology of the People's Republic of China [(2006)398]. The maintenance of mice infected with *S. japonicum* was approved by the Ethics Committee of the National Institute of Parasitic Diseases, Chinese Center for Disease Control and Prevention, Shanghai, China (ref no. 20100525-1).

### Preparation of parasites and protein lysates

Specific pathogen-free female C57BL/6 mice were purchased from the Shanghai Experimental Animal Center, Chinese Academy of Sciences. Female C-57 mice (18–20 g) were randomly divided in to two groups. Both were infected with approximately 60 *S. japonicum* cercariae (Anhui isolate) per mouse and maintained under the same conditions. At 18 dpi, 8 mice of the first group were euthanatized and paired schistosomula were collected. Female and male worms were separated from each other. One Hundred female worms and 100 female worms were collected as 18 dpi females and 18 dpi males samples, respectively. At 28 dpi, four mice from the second group were euthanatized and adult schistosomes were collected as in 18 dpi samples. Forty female worms and 40 female worms were collected as 28 dpi females and 28 dpi males samples. Each sample was divided into two replicates for subsequent analysis.

Parasites were washed twice with cold phosphate-buffered saline (PBS), lysed in lysis buffer [20 mM tris (pH 7.4), 25% glycerol, 1.5 mM MgCl2, 0.2 mM EDTA, 1.2 M KCl] supplemented with Protease Inhibitor Cocktail (Roche, Basel, Switzerland) and HDAC inhibitors (50 mM nicotinamide, 5 μM Trichostatin A), and homogenized by tissue grinding apparatus for 5 min. The homogenates were centrifuged for 10 min at 4°C at 1,000 g. The supernatants were collected and incubated for 15 min, then centrifuged for 30 min at 4°C at 12,000 g. Supernatants were collected again. The protein concentration of each sample was determined by using BCA protein assay kit, and adjusted into 1 mg/ml. Each protein lysate containing 1 mg protein was reduced with 5 mM dithiothreitol (DTT) for 30 min at 56°C, and then alkylated with 15 mM iodoacetamide for 30 min at room temperature in the darkness. The reaction was terminated with cysteine (dissolved in acid condition) at room temperature for 30 min. Ice-cold acetone was added to the samples to completely precipitate the protein at −20°C overnight. The precipitate was freeze-dried, and digested with trypsin (Promega, Madison, USA) at a ratio of 1:50 (enzyme to substrate) at 37°C for at least 12 h. To ensure that the protein was digested completely, additional trypsin was added at a ratio of 1:100 (enzyme to substrate) for additional 3 h. The digested peptides were freeze-dried again.

### Enrichment of lysine acetylated peptides

The dried peptides were dissolved in IP buffer, and incubated with pan anti-acetyllysine antibody conjugated agarose beads (PTM Biolabs, Chicago, USA) at 4°C overnight with gentle shaking. After incubation, the peptides-bound agarose beads were carefully washed three times with Wash Buffer I, once with Wash Buffer II and twice with water. Lysine-acetylated peptides were eluted from the beads with elution buffer and were vacuum dried.

### LC-ESI-MS/MS analysis

The dried peptides were resuspended in 0.1% formic acid. Liquid chromatography electrospray ionization tandem mass spectrometry (LC-ESI-MS/MS) analysis were performed by LTQ-Orbitrap Elite mass spectrometer (Thermo Electron, Bremen, Germany) connected with EASY-nLC 1,000 system (Thermo Fisher Scientific, Sunnyvale, CA, USA). The samples were loaded onto Acclaim PepMap100 C18 trap column (5 μm, 100 Å, 100 μm i.d. x 2 cm, Thermo Fisher Scientific) and separated on an Acclaim PepMap RSLC C18 analytical column (2 μm, 100 Å, 75 μm i.d. x 25 cm, Thermo Fisher Scientific). The peptides were eluted over 40 min by a gradient of 5–35% (1% formic acid in acetonitrile) at a rate of 200 nL/min, and maintained at 90% for 5 min. Intact peptides were detected by FTMS with a resolution of 240,000, while the ion fragments were detected by Ion Trap. MS/MS analysis were performed via a data-dependent procedure, and the top 15 intense precursor ions were selected from one MS scan for a collision-induced dissociation (CID) analysis. For MS scan, the m/z scan range was 350–1,800.

### MS data processing

The raw data were analyzed using Proteome Discover (Version 1.4, Thermo Fisher Scientific) and Mascot server (Version 2.3, Matrix Science, London, UK) search engine. Peptides were matched with the *S. japonicu*m protein V4 database (LSBI-Sjr: 12657 sequences, 4929382 residues), downloaded from the Chinese National Genome Center at Shanghai (http://lifecenter.sgst.cn/schistosoma/). Trypsin/P was used as cleavage enzyme with 2 maximum missed cleavage sites. The mass error was set to 10 ppm for precursor ions and 0.5 Da fragments ions. Carbamidomethylation of cysteine was specified as fixed modification, and oxidation of methionine, acetylation of lysine, acetylation of protein N-term, the conversion of glutamine to pyroglutamate at peptide N-term were used as variable modifications. The false discovery rate (FDR) threshold for proteins, peptides, and modification sites was set to 0.05. Minimum peptide length was set as six amino acids, and the other parameters were set to the default values.

### Bioinformatic analysis

Gene ontology (GO) annotations of the proteins identified with BLAST result were performed with Uniprot Database (http://www.Uniprot.org). GO cluster analysis were conducted by Blast2go 2.8 (https://www.blast2go.com). Acetylated proteins were categorized into biological process, cellular component and molecular function. Pathway annotation was performed with DAVID bioinformatics resources v6.7 (https://david.ncifcrf.gov/summary.jsp), list of GI accessions of acetylated proteins were uploaded to the website and compared with the data of *Homo sapiens*. Pathway enrichment was generated by Kyoto Encyclopedia of Genes and Genomes (KEGG). Subcellular localization of acetylated proteins was extracted from WoLFPSORT (http://www.genscript.com/wolf-psort.html). The enrichment analysis of the 13-mers amino acids, containing six amino acids flanking the acetylated site on each side, was performed by motif-x (http://motif-x.med.harvard.edu/motif-x.html), and same-sized peptides of all proteins in our database were used as the background parameters. Heatmaps were produced by R v3.3.3, with 10 amino acids flanking by both sides of the acetylated sites.

## Results and discussion

### Global view of lysine acetylation in *S. japonicum*

**Figure 1:**
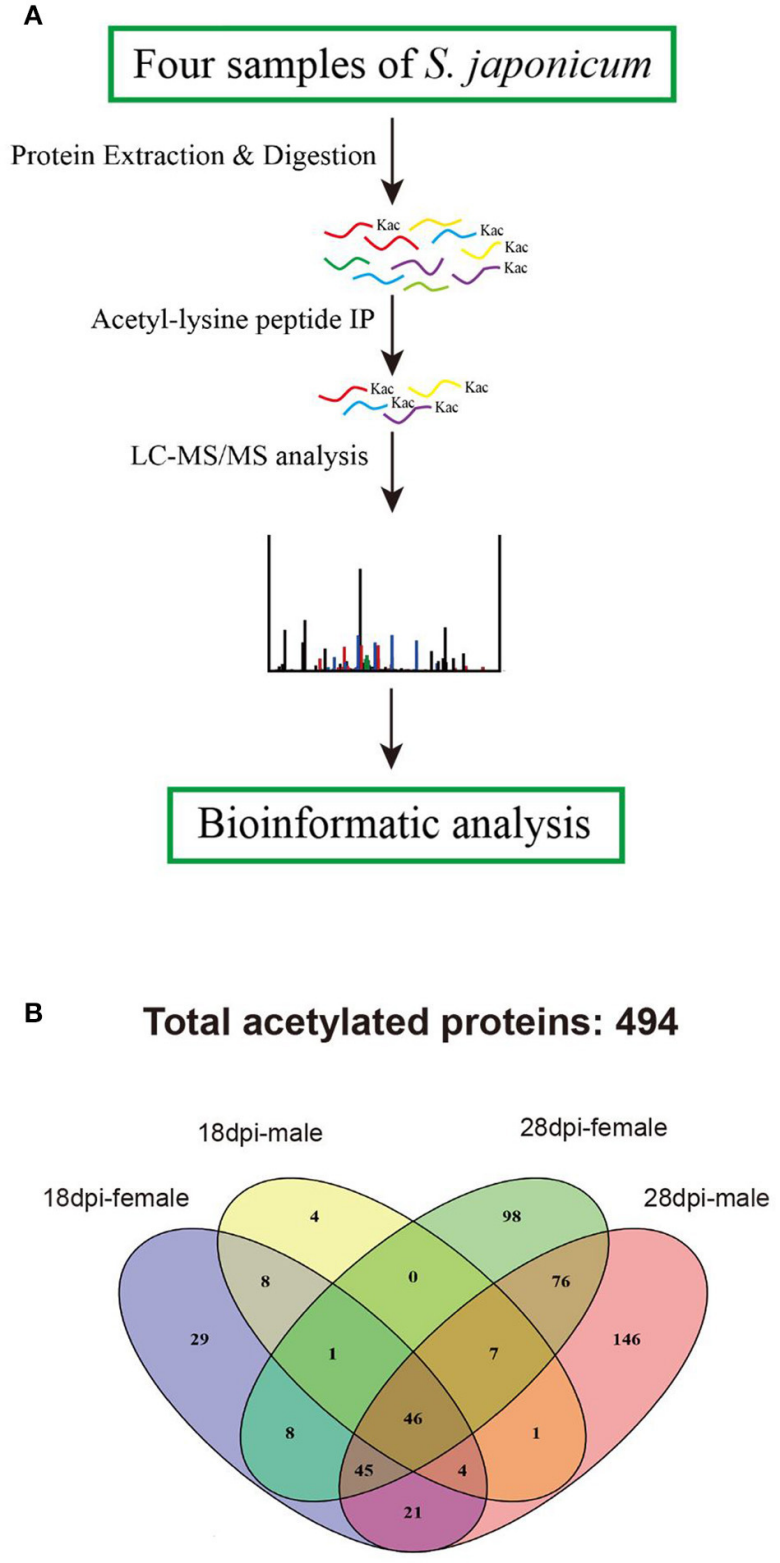
Proteomic analysis strategy to profile the acetylated protein components of *S. japonicum*. **(A)** Overview of analytical procedures used in this study; **(B)** Venn diagram showing the distribution of the acetylated proteins identified from the four samples.

To identify *S. japonicum* acetylome, proteins of different samples were extracted, digested with trypsin to peptides, and the peptides were enriched with pan anti-acetyllysine antibody. Then the enriched acetylated peptides were analyzed by LC-ESI-MS/MS, and the obtained MS/MS spectra were used to search against *S. japonicum* database with MASCOT search engine (Figure 1A). In this study, we identified a total of 874 acetylated sites in 494 proteins in the four samples of 18 dpi females, 18 dpi males, 28 dpi females, 28 dpi males. All acetylated proteins and the corresponding acetylated peptides were listed in Table S1. In 28 dpi males, 616 acetylated sites and 346 acetylated proteins were identified, which were the largest numbers of the four samples. In 18 dpi females, 18 dpi males and 28 dpi females, 231, 102, 430 acetylated sites and 162, 71, 281 acetylated proteins were identified, respectively (Table 1). The four samples shared 47 acetylated sites and 46 acetylated proteins (Figure 1B). The shared proteins consisted of histones (Figure S1), metabolic enzymes, nuclear import proteins, cytoskeleton proteins, proteins related to transcription, pre-mRNA processing and translation, etc., (Table S2). The functions of the shared proteins were consistent with the previous published surveys related to acetylated proteins in *S. japonicum* and in other species (Kouzarides, 2000; Hong et al., 2016). More acetylated sites and proteins were indentified in *S. japonicum* by Hong et al. in a previous study (Hong et al., 2016). This might be attributed to the usage of of *S. japonicum* samples at different stages (42 vs. 18 dpi and 28 dpi), as well as the different tryptic digestion and immunoprecipitation enrichment protocols of the two studies.

**Table 1:** Acetylated sites and acetylated proteins of different *S. japonicum* samples.

**Samples**	**No. of acetylated sites**	**No. of acetylated proteins**
18 dpi female	231	162
18 dpi male	102	71
28 dpi female	430	281
28 dpi male	616	346
Total	874	494

### Acetylation motifs and sites

**Figure 2:**
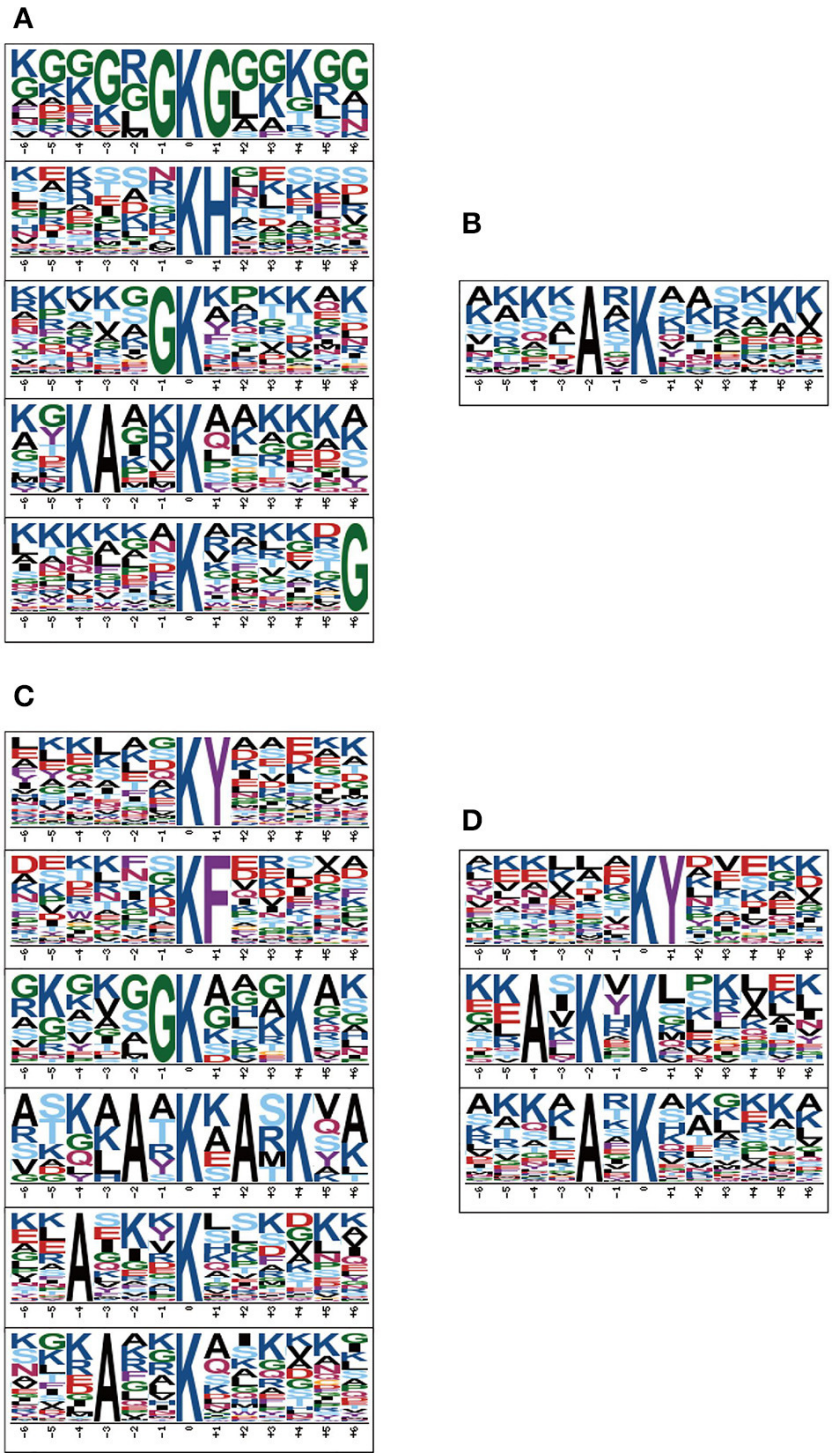
Relative abundance of each amino acid residues surrounding sites of acetylated lysines in proteins of four samples. **(A)** 18 dpi female; **(B)** 18 dpi male; **(C)** 28 dpi female; **(D)** 28 dpi male. The amino acids in specific positions of acetylated lysine (6 amino acids upstream and down stream of the acetylation site) were identified by Motif-X software.

To obtain the conserved motifs of acetylated peptides, we analyzed a set of patterns with acetylated lysine as the center through the Motif-X software. The results showed that 28 dpi female worm had the largest categories of motifs, XXXXXX**KY**XXXX, XXXXXX**KF**XXXXX, XXXXX**GK**XXXKXX, XXXX**A**X**K**X**A**X**K**XX, XX**A**XXX**K**XXXXXX and XXX**A**XX**K**XXXXXX, whereas 18 dpi males had only one motif XXXX**A**X**K**XXXX (Figure 2). Our results showed that the XXX**A**X**K**XXXX motif was conserved in the males as it was detected both in the 18 and 28 dpi males. Besides, the XXXXXX**KY**XXXXX motif was conserved in the adult worms as it was detected both in 28 dpi females and 28 dpi males. Our results were largely in consistent with the findings of previous study except that the **K**XXX**R** motif was not enriched in our study (Hong et al., 2016). In previous study, 42 dpi worms which contained many eggs in females were used. We speculated that the eggs might have acetylation motifs different from those in 18 and 28 dpi worms and thus contributed to the differences between the two studies.

**Figure 3:**
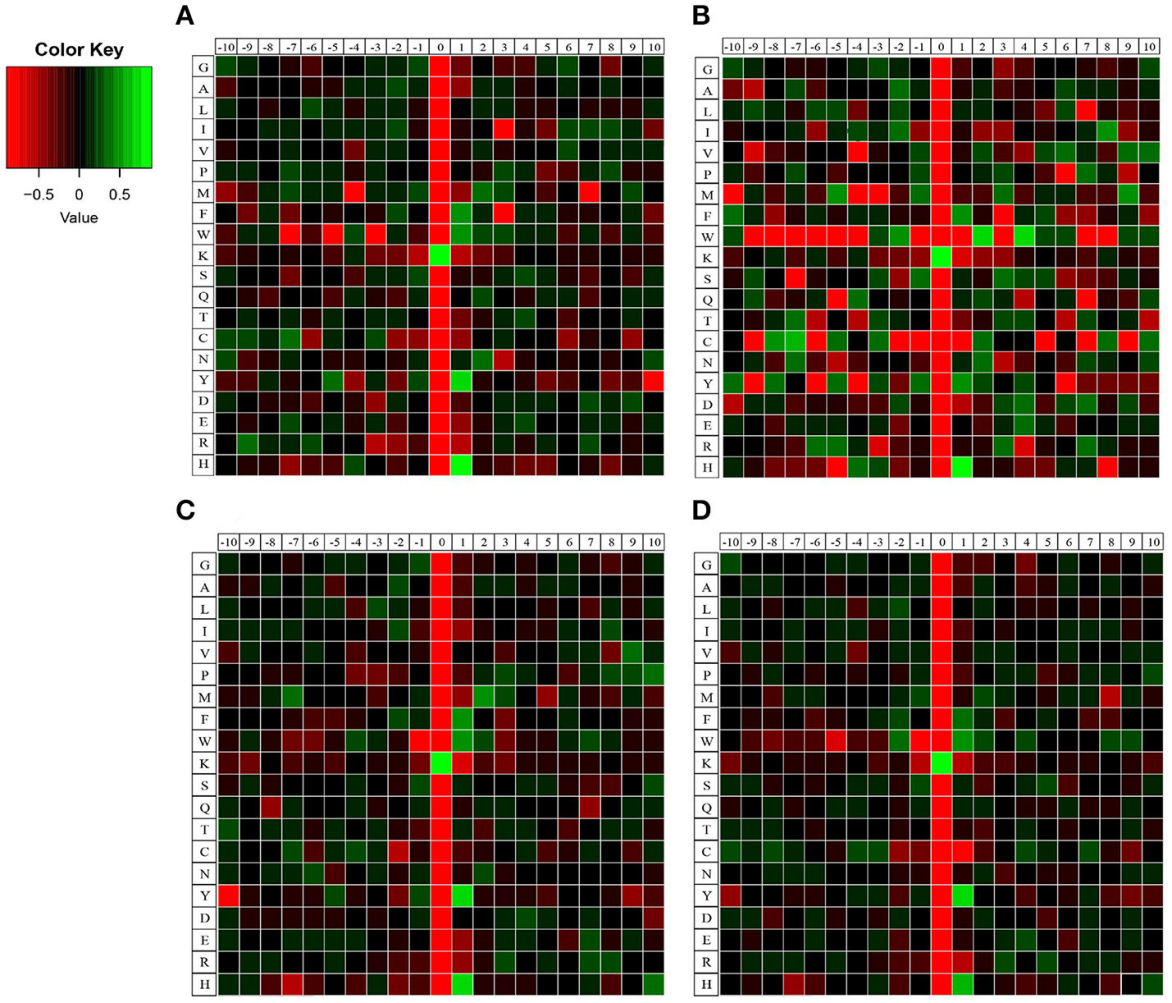
Heatmaps of amino acid compositions of acetylation sites in four samples. **(A)** 18 dpi female; **(B)** 18 dpi male; **(C)** 28 dpi female; **(D)** 28 dpi male. Amino acids that are significantly enriched (green) or depleted (red) compared to the general amino acid composition were displayed.

To elucidate the preference of amino acids surrounding the acetylated lysines, we also generated heatmaps to access whether there was a significant enrichment of specific amino acids. As shown in Figure 3, lysine-acetylated peptides from the four samples exhibited the same preference in their flanking sequence, namely Y (tyrosine) and H (histidine) at +1 position, bearing striking resemblance to those of acetylated proteins in Human cells, *Toxoplasma* and *Escherichia coli* (Kim et al., 2006; Zhang et al., 2009; Jeffers and Sullivan, 2012). However, different from other samples, there is a unique preference for W at the +2 and +4 position in 18 dpi females. Besides, different levels of enrichment of other amino acids were also detected, which indicated that lysine-acetylated modification might be catalyzed by subunits of acetyltransterases with preferred substrates.

### Number of acetylation sites

**Figure 4:**
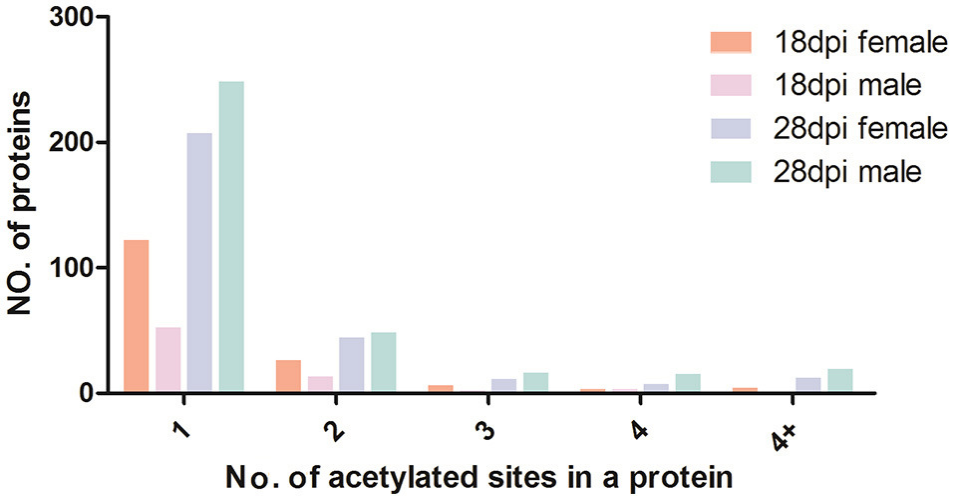
Distribution of acetylated proteins based on the numbers of acetylation sites.

We calculated the number of acetylated sites in each protein of the four samples. Although over 60% of the acetylated proteins of each sample contained only one acetylated site, many other proteins also contained multiple acetylated sites (Figure 4). The multiple lysine-acetylated sites in one protein indicated this protein might have versatile molecular functions. Proteins with multiple acetylated sites were more frequently identified in 28 dpi females and males (74 and 98 proteins, respectively) than in 18 dpi females and males (29 and 19 proteins, respectively, Table S3). Among the highly acetylated proteins, we sorted out proteins with over four acetylated sites, and found that histone proteins were most likely to have multiple acetylated sites in 18 dpi females and males. However, Sjc_0054830 (ko: K10352 myosin heavy chain, putative) was identified with 27 actylated sites in 28 dpi male, which was the protein with most acetylated sites in all samples. Myosin are responsible for actin-based motility and muscle contraction (Houdusse and Sweeney, 2016). Previous studies also showed that lysine acetylation of myosin enhanced its enzymatic and motor activity by decreasing the Km for that actin-activated ATPase activity of myosin heavy chain isoforms to increase contractile performance (Gupta et al., 2008; Samant et al., 2015). We speculated the high acetylation level of myosin might be associated with the need of strong muscles, to maintain male-female pairing and the attachment to venous wall with suckers. Moreover, in 28 dpi females and males, many metabolic enzymes, such as 6-phosphofructokinase, enolase, and malate dehydrogenase, also contained multiple acetylated sites.

### Subcellular localization

**Figure 5:**
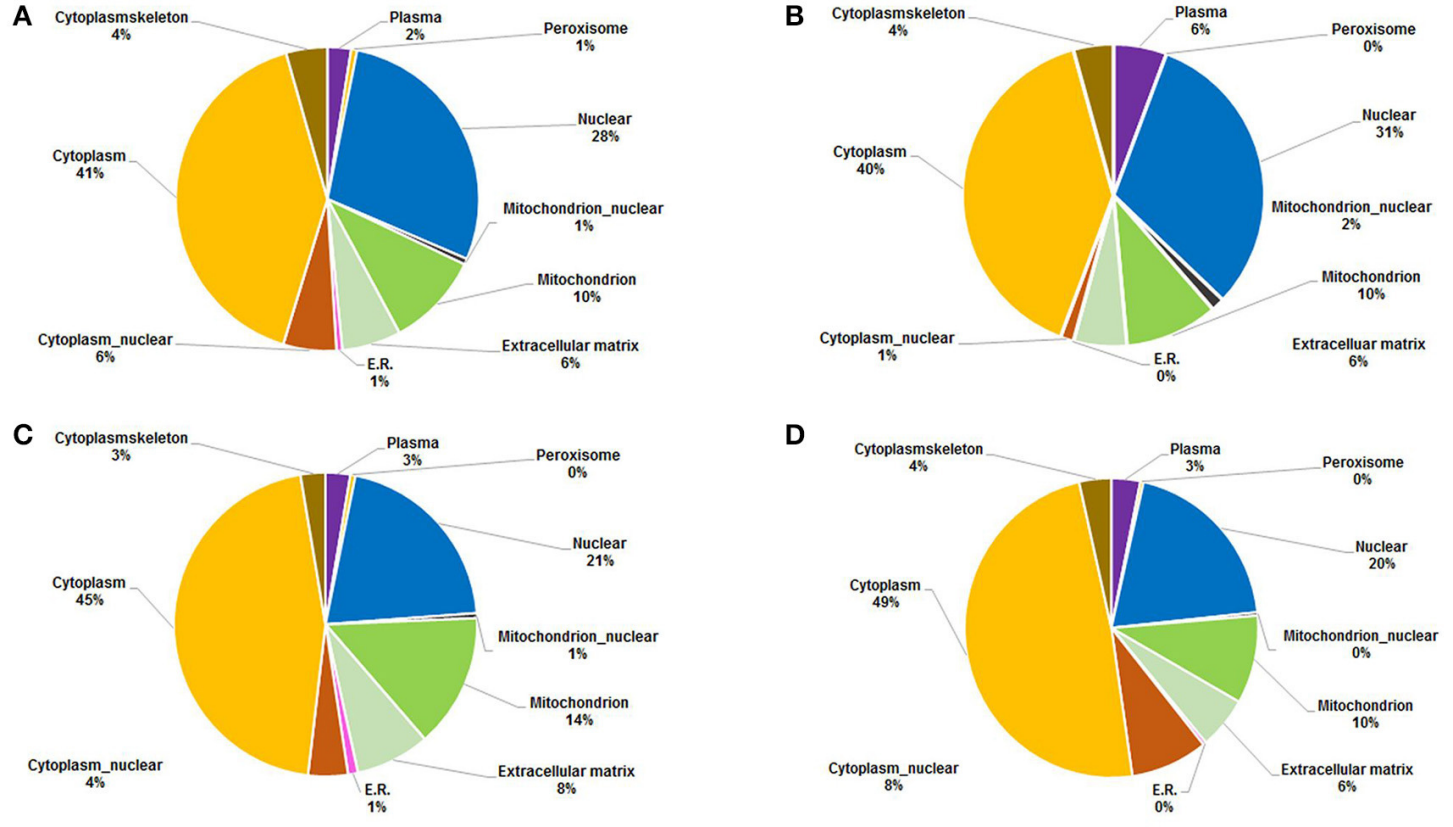
Subcellular localization of acetylated proteins in four samples. **(A)** 18 dpi female; **(B)** 18 dpi male; **(C)** 28 dpi female; **(D)** 28 dpi male.

The subcellular localization of acetylated proteins of the four samples was analyzed and the results showed that nearly 70% of the acetylated proteins were located in the cytosol and nuclear. A small part of acetylated proteins were localized in extracellular, cytoskeletal, and endoplasmic reticulum (Figure 5). The most acetylated proteins were localized in cytoplasmic, accounting for 41, 40, 45, and 49% in 18 dpi females, 18 dpi males, 28 dpi females and 28 dpi males, respectively. Previous studies showed that the most abundant acetylated cytoplasmic proteins were proteins involving in metabolism (Sadoul et al., 2011; Miao et al., 2013). Our data also suggested that acetylation mainly happened in the proteins related to metabolism in both developmental stages. The increased percentages of cytoplasmic acetylated proteins in 28 dpi worms suggested they might be associated with the more active metabolism in adult worms than in juvenile worms. The second most abundant acetylated proteins were localized in the nucleus. The percentages of nuclear acetylated proteins in 18 dpi male and females (28, 31%) were larger than in 28 dpi males and females (21, 20%). As most of the acetylated nuclear proteins were histones and transcription-related proteins, we speculated that at 18 dpi, schistosomula focused more on the regulation of gene transcription in nucleus. The third most abundantly acetylated proteins were localized in mitochondria. Mitochondrial acetylated proteins accounted for 14% of all acetylated proteins in 28 dpi females and 10% in the other three samples. A previous study showed that over one-third of all proteins in mitochondria were acetylated, and the majority of mitochondrial acetylated proteins were involved in energy metabolism (Anderson and Hirschey, 2012). Since 28 dpi females are sexually mature and start laying eggs, a higher energy metabolism is required.

### Functional annotations of acetylated proteins

**Figure 6:**
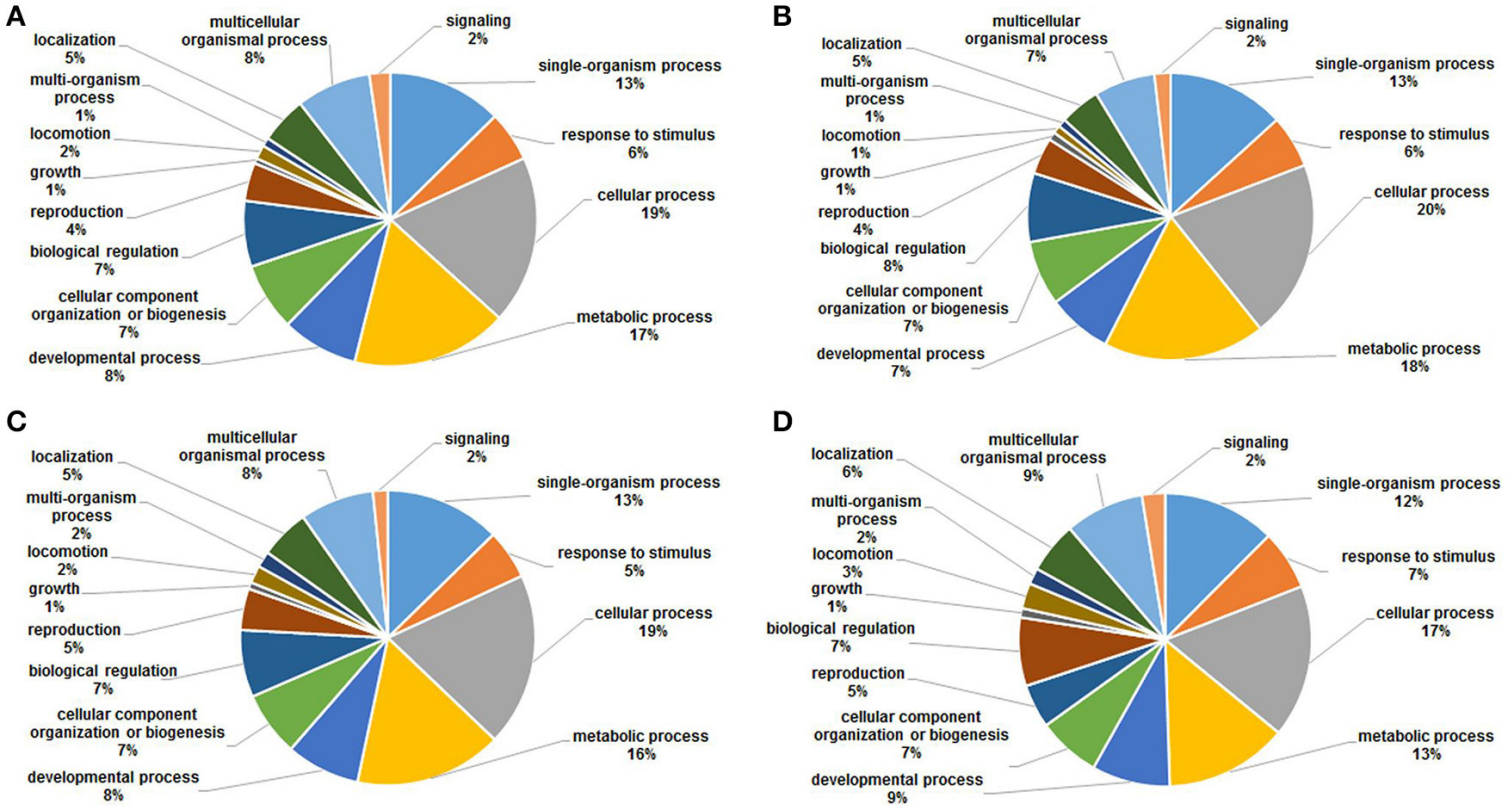
GO functional classification of the identified acetylated proteins based on biological processes in four samples. **(A)** 18 dpi female; **(B)** 18 dpi male; **(C)** 28 dpi female; **(D)** 28 dpi male.

To gain a better understanding of the functions and distributions of the lysine acetylated proteins, GO annotation was performed. Biological process analysis showed that proteins related to cellular process (19, 20, 19, and 17%), metabolic process (17, 18, 16, and 13%) and developmental process (13, 13, 13, and 12%) were the major acetylated proteins in the four samples (Figure 6). Previous studies showed that certain proteins associated with metabolism and cellular process could be acetylated and this reversible acetylated modification emerged as an important regulatory mechanism in cellular metabolism in human, bacteria and animal (Zhao et al., 2010; Scott, 2012; Xiong and Guan, 2012). Therefore, in this study, we speculated that lysine acetylation might also play vital roles in metabolism and cellular regulation during the development of *S. japonicum*.

**Figure 7:**
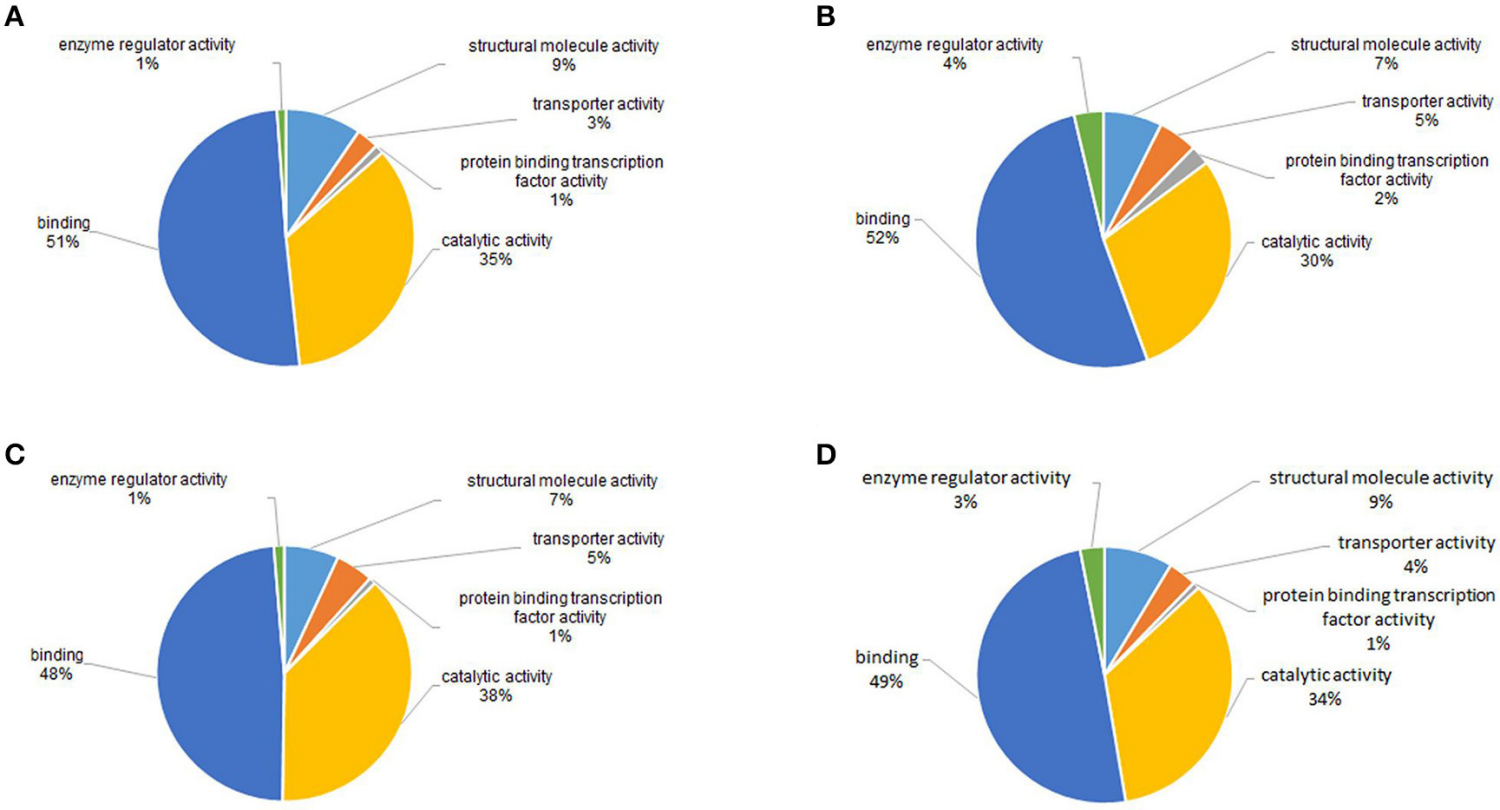
GO functional classification of the identified acetylated proteins based on molecular function in four samples. **(A)** 18 dpi female; **(B)** 18 dpi male; **(C)** 28 dpi female; **(D)** 28 dpi male.

Cellular component analysis showed that the acetylated proteins belonged to many cellular components, and the proportions of each component did not exhibit significant differences except for the acetylated proteins in membrane (Figure 7). Membrane acetylated proteins in 18 dpi females and males accounted for only 6 and 5% of all acetylated proteins, while the percentages in 28 dpi females and males were 9 and 10%. This suggested that signal transduction and molecule transporting were more active in adults than in juveniles.

**Figure 8:**
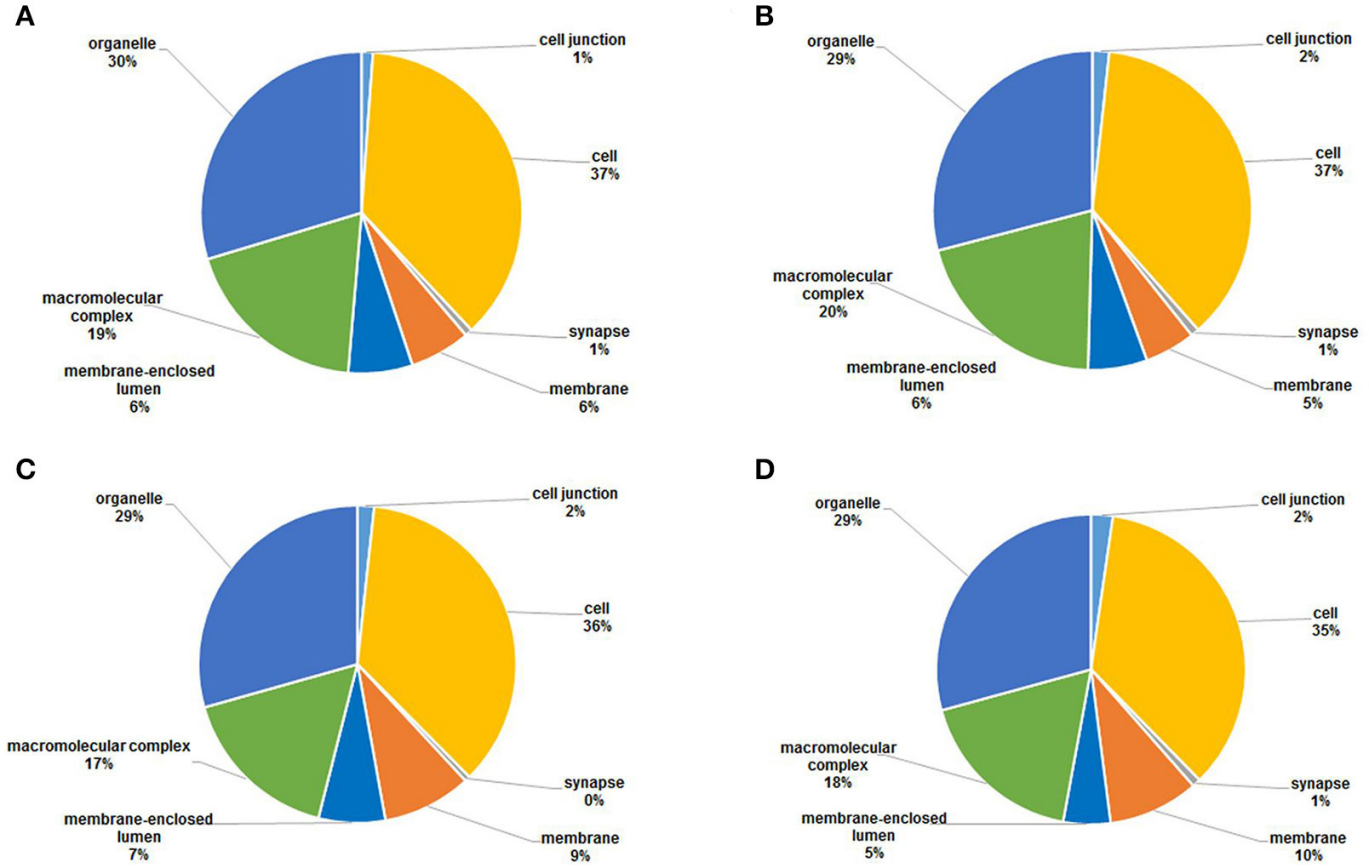
GO functional classification of the identified acetylated proteins based on cellular components in four samples. **(A)** 18 dpi female; **(B)** 18 dpi male; **(C)** 28 dpi female; **(D)** 28 dpi male.

Molecular function analysis showed that proteins with catalytic activity and binding activity were the two largest groups of acetylated proteins, which accounted for nearly 85% of all the proteins in all four samples (Figure 8). This was in accordance with the studies in plant and bacteria (Fang et al., 2015; Xie et al., 2015). Previous studies showed that nearly all enzymes involved in glycolysis, gluconeogenesis, tricarboxylic acid (TCA) cycle, fatty acid oxidation, urea cycle, nitrogen metabolism and glycogen metabolism could be acetylated (Guan and Xiong, 2011). Therefore, catalytic and binding activities were the most important molecular functions of acetylated proteins.

### Functional enrichment of acetylated proteins

To determine which type of proteins was more likely to be acetylated, GO terms enrichment analysis were performed. The top10 significant GO terms in biological process and molecular function and the top 5 in cellular component were enriched according to their *p*-value and were displayed in Figure S2. In the biological process category, the most GO terms in all samples focused on the assembly of various macromolecules and protein-complexes, including histones, myosin, tubulin, translation initiation factors, etc., We speculated that acetylation had an important impact on the assembly or disassembly of these macromolecules, thus influenced biological functions of *S. japonicum*. Differences were also found between samples. In 18 dpi females, no GO terms related to carbohydrate metabolism were enriched; whereas in 28 dpi females, a number of GO terms related to carbohydrate metabolic process were enriched, including monosaccharide catabolic process, hexose metabolic process, glucose metabolic process, etc. This might suggest that carbohydrate metabolism in 28 dpi females was more active than in 18 dpi females to fulfill the increased energy and nutrition demand for egg production. Although, similar numbers of GO terms related to carbohydrate metabolism were enriched in 18 and 28 dpi males, the specific annotations were different. In 18 dpi males, carbohydrate catabolic process, glycolysis, and glucose metabolic process was enriched; whereas in 28 dpi males, monosaccharide catabolic process and glucose metabolic process was enriched. The different enriched terms related to carbohydrate metabolism suggested that pathways of energy generation from carbohydrates in juvenile and adult males might be distinct as different types of carbohydrates and metabolic processes were involved.

In the molecular function category, the most GO terms in all samples focused on various binding activities, except for 18 dpi males in which only one molecular function was enriched. Compared with 18 dpi males, a number of GO terms which might associate with cytoskeleton proteins were enriched, including microfilament motor activity, actin binding, cytoskeletal protein binding, and actin filament binding. As acetylation of actin promotes muscular movement (Li and Yang, 2015), this indicated that muscle contraction and movement in adult males were more active than in juvenile males.

In the cellular component category, GO terms enriched in 18 dpi males and females were mainly endonuclear, including chromosome, nucleosome, and DNA packaging complex, etc., Whereas, in 28 dpi males and females, the enriched GO terms were mainly cytoplasmic, including cytoplasm, intracellular, and cytoplasmic part. The different enrichments might reflect that at the schistosomula stage, cells proliferated rapidly and focused on endonuclear activities, such as DNA replication and transcription; while the adult worms focused on egg laying, thus cytoplasmic activities, such as metabolism and protein synthesis were more active.

### KEGG analysis

**Figure 9:**
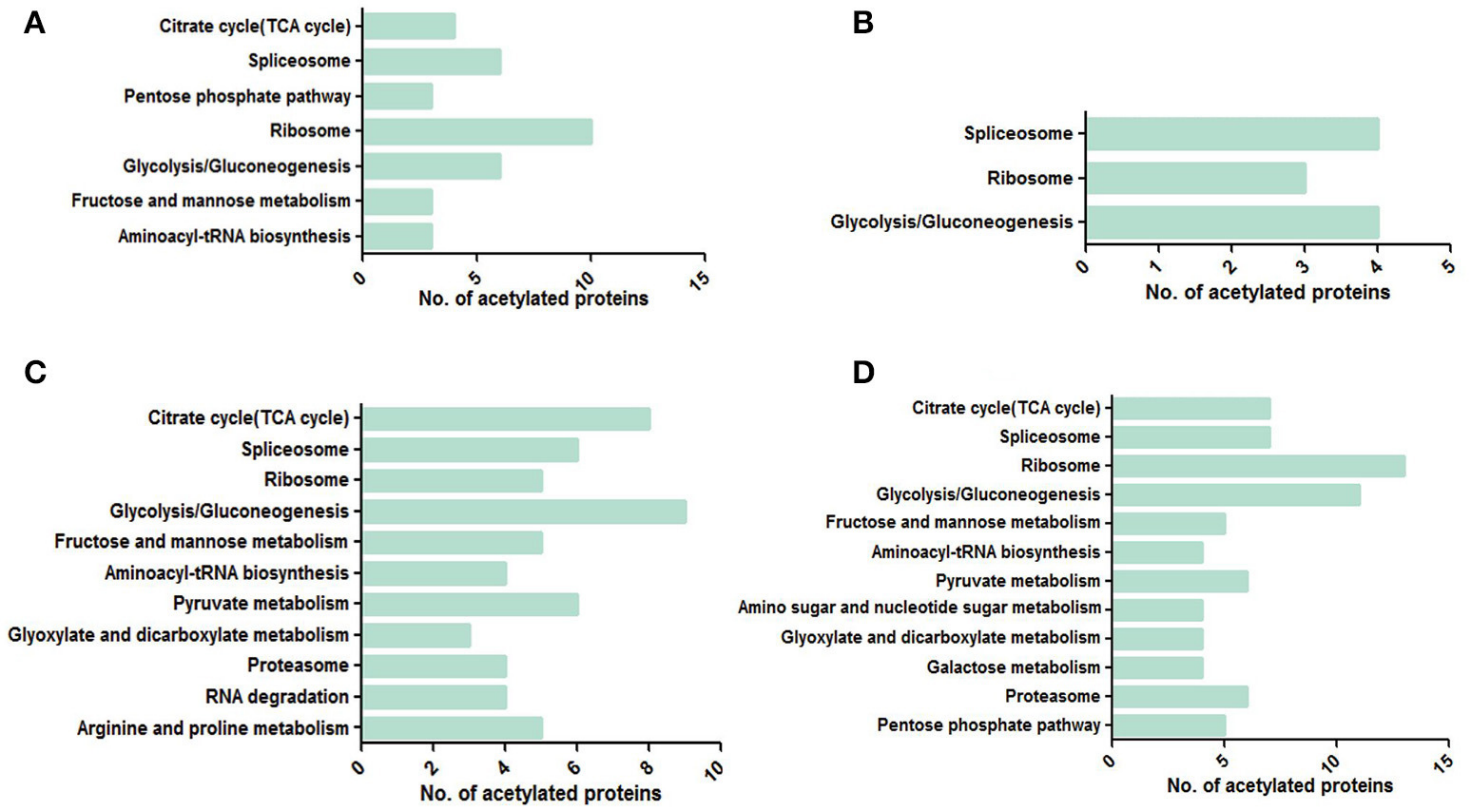
KEGG enrichment pathway analyses in four samples. **(A)** 18 dpi female; **(B)** 18 dpi male; **(C)** 28 dpi female; **(D)** 28 dpi male.

Kyoto Encyclopedia of Genes and Genomes (KEGG) pathway analysis showed that the acetylated proteins were enriched in the pathways of splicesome, ribosome and glycolysis/gluconeogenesis in all four samples, indicating that acetylation had a potential impact on pre-mRNA processing, protein biosynthesis and the regulation of cellular metabolism especially glycometabolism (Figure 9). Moreover, more metabolic pathways were enriched in 28 dpi worms than in 18 dpi worms. In females, the pathways of pyruvate metabolism, proteasome, arginine and proline metabolism were specifically enriched in 28 dpi. This suggested that carbohydrate and protein digestion was increased in adult females to fulfill their increased energy and nutrition demand for egg laying. Similarly, in males, pathways related to carbohydrate metabolism and TCA cycle were specifically enriched in 28 dpi, also suggesting that metabolism was more active in adult males.

### Lysine acetylation associated with *S. japonicum* development

Analysis showed that acetylated proteins mainly associated with the following molecular functions: protein binding, organic cyclic compound binding, heterocyclic compound binding, and ion binding (Table 2). It was also shown that the numbers of acetylated proteins changed drastically between juvenile and adult males. Only 53 acetylated protein-binding proteins were identified in 18 dpi males, whereas 124 were identified in 28 dpi male. Similarly, 69 and 125 organic cyclic compound binding proteins were acetylated in 18 and 28 dpi males, respectively. Moreover, the numbers of other binding-related proteins, oxidoreductase activity proteins and transmembrane transporter activity proteins in 28 dpi males were more than twice that of 18 dpi males. These changes might associate with the biological changes after female-male pairing. However, during sexual maturation, the numbers of acetylated proteins in females change less drastically than in males, and no distinct differences on molecular functions were exhibited.

**Table 2:** List of molecular function enrichment of acetylated proteins in all samples.

**Functions**	**No. of acetylated proteins**			
	**18 dpi♀**	**28 dpi♀**	**18 dpi♂**	**28 dpi♂**
Protein binding	49	66	53	124
Organic cyclic compound binding	74	69	79	125
Heterocyclic compound binding	73	68	78	124
Ion binding	55	65	59	126
Structural constituent of ribosome	11	6	11	17
Small molecule binding	49	53	52	95
Carbohydrate derivative binding	41	39	43	80
Transferase activity	16	16	17	36
Ligase activity	9	6	9	13
Lipid binding	4	8	5	15
Structural constituent of cytoskeleton	3	3	3	6
Structural constituent of muscle	3	3	4	4
Hydrolase activity	29	43	31	68
Oxidoreductase activity	7	15	7	21
Chromatin binding	3	1	3	4
Cofactor binding	3	9	3	11
Metal cluster binding	4	4	4	4
Signal transducer activity	4	–	4	4
Transcription factor binding	2	2	2	3
Sulfur compound binding	1	3	1	5
Sequence–specific DNA binding	1	–	1	3
Enzyme inhibitor activity	1	2	1	3
Substrate–specific transporter activity	4	9	4	10
Carbohydrate binding	1	–	1	1
Isomerase activity	3	7	3	9
Lyase activity	3	6	3	6
Small protein activating enzyme activity	1	–	1	1
Drug binding	1	–	1	1
Transmembrane transporter activity	3	8	3	9
Enzyme activator activity	–	–	1	1
Nucleoside–triphosphatase regulator activity	–	1	1	5
Peptidase regulator activity	–	1	–	1
Kinase regulator activity	–	–	–	3
Calcium channel regulator activity	–	–	–	1
Channel inhibitor activity	–	–	–	1

*♀, Female; ♂, Male*.

To further investigate how acetylation related to biological changes during development, acetylated proteins were classified according to their biological functions, and they were compared between the 18 and 28 dpi stage (Table 3). Our results showed that in 28 dpi adults, more proteins related to glycometabolism and lipid metabolism, transporting and antioxidation were acetylated than in 18 dpi schistosomula. Acetylated modification is a mean to regulate intracellular metabolic network. Enzymes associated with glycometabolism, lipid acid oxidation, and urea cycle could be regulated by acetylated modification (Guan and Xiong, 2011). The enrichment of acetylated enzymes in adult worms might be associated with the increased metabolic activities to fulfill the increased energy demand after sexual maturation (Pearce and Huang, 2015). Moreover, It has been reported that protein acetylation promoted transporting activity in cytoplasm (Sadoul et al., 2011). We speculated that in adult worms, acetylation of transport-related proteins might also enhance their transport activity, which also indicated that metabolism in adult worms was more active than in schistosomula. Furthermore, residing in hepatic portal venous system of the host, adult schistosomes exhibited stronger anti-oxidant activity than schistosomula, to protect them from the free oxygen produced by digestion of erythrocytes. We speculated that in *S. japonicum*, acetylation of antioxidant proteins was associated with the increased antioxidant activity in adults (LoVerde, 1998; Heather and Alger, 2002).

**Table 3:** Biological function clusterings of stage-specific acetylated proteins in all samples.

**Function group**	**No. of acetylated proteins**			
	**18 dpi♀**	**28 dpi♀**	**18 dpi♂**	**28 dpi♂**
Glucose metabolism	2	3	1	5
Regulation of tanscription and translation	4	6	0	4
Cell proliferation	3	3	0	3
Immune suppression	3	0	0	0
Muscular movement	3	0	0	9
Antioxidant	0	3	1	7
Lipid metabolism	0	2	0	3
Transport (protein, molecular)	0	6	0	9
Energy metabolism	0	6	0	5
Protein degradation	0	6	1	3
Signaling	0	0	1	10
Environmental stress resistance	0	0	0	4

*♀, female; ♂, male*.

### Acetylation associated with biological changes during female development

The stage-specifically acetylated proteins in 18 and 28 dpi females were compared and their biological functions were analyzed (Tables 4, 5). Firstly, more cytoskeleton proteins (e.g., actin-2, tubulin alpha) in 18 dpi females were acetylated than in 28 dpi females. Both actin and tubulin are cytoskeleton proteins involving in cell motility and cell shape maintenance. High-leveled acetylation of tubulin enhances microtubule stability and modulates immuno-stress (Sadoul et al., 2011). Acetylation of actin not only promotes cellular movement, intracellular transport, and transcriptional regulation, but also accelerates its interaction with myosin and promotes muscular movement (Li and Yang, 2015). Therefore, the different acetylation status of actin and tubulin in different developmental stages indicated a decline of muscular activity in 28 dpi females compared to 18 dpi females. In 18 dpi, only a fraction of females are paired with males. Therefore, the movement of females still rely on their own muscles. Whereas, in 28 dpi, most males and females are paired and the movement is dominated by males, thus the female muscles are less required for movement.

**Table 4:** Biological function clustering of acetylated proteins specific in 18 dpi females.

**Functions**	**Protein ID**	**Annotations**
Glucose metabolism	Sjc_0046600	ko:K01187 alpha-glucosidase [EC3.2.1.20], putative
	Sjc_0030250	ko:K00033 6-phosphogluconate dehydrogenase [EC1.1.1.44], putative
Regulation of transcription and translation	Sjc_0019830	SAGA-associated factor 29 homolog, putative
	Sjc_0210040	Similar to Putative U5 small nuclearear ribonucleareoprotein 200 kDa helicase, putative
	Sjc_0208180	ko:K09497 T-complex protein 1 subunit epsilon, putative
	Sjc_0068070	Ufm1-conjugating enzyme 1, putative
Cell proliferation	Sjc_0005910	ko:K01919 glutamate–cysteine ligase [EC6.3.2.2], putative
	Sjc_0303290	Cysteine and histidine-rich domain-containing protein 1, putative
	Sjc_0301340	Far upstream element-binding protein 2, putative
Immune suppression	Sjc_0025140	ko:K09568 FK506-binding protein 1, putative
	Sjc_0303020	ko:K01802 peptidylprolyl isomerase [EC5.2.1.8], putative
	Sjc_0091280	IPR001152, Thymosin beta-4, domain-containing
Muscular movement	Sjc_0002370	Actin-2, putative
	Sjc_0042620	ko:K05692 actin beta/gamma 1, putative
	Sjc_0004280	ko:K07374 tubulin alpha, putative

**Table 5:** Biological function clustering of acetylated proteins specific in 28 dpi females.

**Functions**	**Protein ID**	**Annotations**
Glucose metabolism	Sjc_0211220	IPR006015, Universal stress protein (Usp); IPR006016, UspA, domain-containing
	Sjc_0301250	ko:K01810 glucose-6-phosphate isomerase [EC5.3.1.9], putative
	Sjc_0073030	ko:K00164 2-oxoglutarate dehydrogenase E1 component [EC1.2.4.2], putative
Cell proliferation	Sjc_0094390	IPR002049, EGF-like, laminin; IPR003599, Immunoglobulin subtype;IPR013032, EGF-like region; IPR007110, Immunoglobulin-like, domain-containing
	Sjc_0214350	ko:K06942 OLA1; Obg-like ATPase 1, putative
	Sjc_0088690	Neuroblast differentiation-associated protein AHNAK, putative
Regulation of transcription and translation	Sjc_0214350	ko:K06942 OLA1; Obg-like ATPase 1, putative
	Sjc_0030640	ko:K03687 molecular chaperone GrpE, putative
	Sjc_0031970	Eukaryotic translation initiation factor 3 subunit A, putative
	Sjc_0212210	RNA-binding protein 4, putative
	Sjc_0025200	ko:K03122 transcription initiation factor TFIIA large subunit, putative
	Sjc_0031970	Eukaryotic translation initiation factor 3 subunit A, putative
Antioxidant	Sjc_0038300	ko:K00799 glutathione S-transferase [EC2.5.1.18], putative
	Sjc_0035630	ko:K00871 phosphorylase kinase gamma subunit [EC2.7.11.19], putative
	Sjc_0078530	ko:K01069 hydroxyacylglutathione hydrolase [EC3.1.2.6], putative
Transport	Sjc_0101450	IPR001464, Annexin, domain-containing
	Sjc_0209790	ko:K02150 V-type H+-transporting ATPase subunit E, putative
	Sjc_0211260	Annexin A7, putative
	Sjc_0032940	IPR008387, ATPase, F0 complex, subunit F6, mitochondrial, domain-containing
	Sjc_0071850	Phosphate carrier protein, mitochondrial precursor, putative
	Sjc_0043880	Vacuolar protein sorting-associated protein 13A, putative
Energy metabolism	Sjc_0200590	ko:K00933 creatine kinase [EC2.7.3.2], putative
	Sjc_0049690	ATP synthase subunit O, mitochondrial precursor, putative
	Sjc_0304690	ko:K01803 triosephosphate isomerase (TIM) [EC5.3.1.1], putative
	Sjc_0114080	ko:K00261 glutamate dehydrogenase (NAD(P)+) [EC1.4.1.3], putative
	Sjc_0217510	ko:K01676 fumarate hydratase, class I [EC4.2.1.2A], putative
	Sjc_0054460	ko:K00016 L-lactate dehydrogenase [EC1.1.1.27], putative
Protein degradation	Sjc_0068310	ko:K02736 20S proteasome subunit beta 7, putative
	Sjc_0064130	ko:K06690 20S proteasome subunit alpha 8, putative
	Sjc_0096060	ko:K03036 26S proteasome regulatory subunit N6, putative
	Sjc_0060830	ko:K05610 ubiquitin carboxyl-terminal hydrolase L5, putative
	Sjc_0068410	Cullin-associated NEDD8-dissociated protein 1, putative
	Sjc_0214180	Cathepsin B-like cysteine proteinase precursor, putative

Secondly, fatty acid-binding protein 7 and glycerol-3-phosphate dehydrogenase, which associated with fatty acid metabolism, were acetylated in 28 dpi males, whereas no such acetylated proteins were detected in 18 dpi males. Glycerol-3-phosphate dehydrogenase links the fatty acid metabolic pathway with TCA cycle, so that energy can be generated via oxidative phosphorylation. Female egg production requires a large amount of energy. Besides glucose, lipids also serve as an indispensable source for energy and nutrition (Pearce and Huang, 2015). More lipids are reserved for egg laying in mature female vitelline glands than in schistosomula (Huang et al., 2012). When fatty acid metabolism is inhibited in schistosomes, mature females fail to lay eggs. Reversely, when the host is fed with a lipid-rich diet, the fecundity of females increased accordingly (Stanley et al., 2009; Huang et al., 2012). Protein acetylation has been reported to be associated with modulation of fatty acid metabolism (Guan and Xiong, 2011). Therefore, acetylation of proteins associated with fatty acid metabolism in 28 dpi females is required to ensure energy supply for egg laying.

Thirdly, compared with 18 dpi females, a number of proteins associated with protein degradation (e.g., proteasome subunit beta 7,ubiquitin carboxyl-terminal hydrolase), were acetylated specifically in 28 dpi females. The degree of protein acetylation is an important factor determining the rate of protein degradation (Caron et al., 2005; Sadoul et al., 2008). Furthermore, acetylation promotes the digestive activities of proteases and other enzymes (Spange et al., 2009). To meet their energy demand for egg production, the digestion of erythrocytes is more active in mature females than in juvenile females. Therefore, acetylation of proteases in 28 dpi females seems to modulate their enzymatic activities.

Fourthly, in 28 dpi females, more proteins associated with energy metabolism, such as creatine kinase and ATP synthase subunit O, were acetylated than in 18 dpi females. This suggested that acetylation participated more in the regulation of energy metabolism in adult schistosome. We inferred due to the energy demand for egg production, energy metabolism is more active in 28 dpi females, and this might be associated with the more acetylated metabolic proteins in our result.

Fifthly, a number of transport-associated proteins, including annexin and vacuolar protein sorting, were acetylated specifically in 28 dpi females. This might be associated with the enhanced molecular/protein transport activities in adult females. Adult females require strong molecular/protein transport activities to fulfill their biological demand, as well as to transport a large amount of energy and nutrition to the ovary and vitelline glands for egg production.

Finally, in 28 dpi females, more proteins associated with antioxidation were acetylated than in 18 dpi females. To meet their nutrition demands for egg laying, 28 dpi adult worms must intake and digest a large quantity of erythrocytes. The free oxygen molecules released from digested erythrocytes might cause oxidative damage to schistosomes. Thus, antioxidant activities might be essential to protect schistosomes from the oxygen-rich environment. Moreover, the antioxidant proteins also involve in host immunity evasion (Ross et al., 2012). In this study, we discovered many more antioxidant proteins that can be acetylated or deacetylated in 28 dpi females, including phosphorylase kinase which had the function of sensing oxygen pressure. Acetylation of these proteins might be essential for schistosome survival.

### Acetylation associated with biological changes during male development

The stage-specific acetylated proteins in 18 and 28 dpi males were compared and their biological functions were analyzed (Tables 6, 7). Firstly, in 28 dpi males, we identified 10 specific acetylated proteins associated with signal transduction, including calcium/calmodulin-dependent protein kinase, cAMP-dependent protein kinase regulator and Rab GDP dissociation inhibitor. In contrast to 28 dpi males, only 1 specific protein associated with signal transduction was acetylated in 18 dpi males. Pairing is known to be essential to stimulate the development of female productive system and egg laying, possibly via signal transduction from males to females (Michaels, 1969; Fried, 1986; Schussler et al., 1997; Wu and Loverde, 2011). Meanwhile, male worms are more responsible for the interaction with host than the female worms when they are paired. Therefore, acetylation of proteins associated with signal transduction in adult males might be associated with these biological processes.

**Table 6:** Biological function clustering of acetylated proteins specific in 18 dpi males.

**Functions**	**Protein ID**	**Annotations**
Glucose metabolism	Sjc_0046600	ko:K01187 alpha-glucosidase [EC3.2.1.20], putative
Antioxidant	Sjc_0042620	ko:K05692 actin beta/gamma 1, putative
Protein degradation	Sjc_0065510	ko:K03178 ubiquitin-activating enzyme E1, putative
Signaling	Sjc_0006130	TBC1 domain family member 15, putative

**Table 7:** Biological function clustering of acetylated proteins specific in 28 dpi males.

**Functions**	**Protein ID**	**Annotations**
Glucose metabolism	Sjc_0211850	ko:K00615 transketolase [EC2.2.1.1], putative
	Sjc_0207460	ko:K00026 malate dehydrogenase [EC1.1.1.37B], putative
	Sjc_0211970	ko:K00850 6-phosphofructokinase [EC2.7.1.11], putative
	Sjc_0301250	ko:K01810 glucose-6-phosphate isomerase [EC5.3.1.9], putative
	Sjc_0201440	ko:K00873 pyruvate kinase [EC2.7.1.40], putative
Regulation of transcription and translation	Sjc_0219200	ko:K03242 translation initiation factor eIF-2 gamma subunit, putative
	Sjc_0057730	Nocturnin, putative
	Sjc_0216870	WW domain-binding protein 11, putative
	Sjc_0001510	ko:K06100 symplekin, putative
Antioxidant	Sjc_0038300	ko:K00799 glutathione S-transferase [EC2.5.1.18], putative
	Sjc_0039090	ko:K03671 thioredoxin 1, putative
	Sjc_0026090	ko:K00036 glucose-6-phosphate 1-dehydrogenase [EC1.1.1.49], putative
	Sjc_0095720	ko:K03386 peroxiredoxin (alkyl hydroperoxide reductase subunit C) [EC1.11.1.15], putative
	Sjc_0030250	ko:K00033 6-phosphogluconate dehydrogenase [EC1.1.1.44], putative
	Sjc_0005910	ko:K01919 glutamate–cysteine ligase [EC6.3.2.2], putative
	Sjc_0008070	ko:K05687 protein DJ-1, putative
Protein degradation	Sjc_0300430	ko:K08585 calpain, invertebrate, putative
	Sjc_0056120	Suppressor of G2 allele of SKP1 homolog, putative
	Sjc_0102150	Similar to Ubiquitin-conjugating enzyme E2-17 kDa, putative
Signaling	Sjc_0093470	Rab GDP dissociation inhibitor alpha, putative
	Sjc_0101350	ko:K01768 adenylate cyclase [EC4.6.1.1], putative
	Sjc_0302840	ko:K04739 cAMP-dependent protein kinase regulator, putative
	Sjc_0211700	ko:K02183 calmodulin, putative
	Sjc_0001030	ko:K01802 peptidylprolyl isomerase [EC5.2.1.8], putative
	Sjc_0092590	ko:K06630 tyrosine 3-monooxygenase/tryptophan 5-monooxygenase activation, putative
	Sjc_0053680	14-3-3 protein beta/alpha-A, putative
	Sjc_0121020	Calcium/calmodulin-dependent protein kinase type II alpha chain, putative
	Sjc_0021650	ko:K03115 casein kinase 2, beta polypeptide, putative
	Sjc_0069470	IPR000198,RhoGAP;IPR008936,Rho GTPase activation protein,domain-containing
Resist environmental pressure	Sjc_0211220	IPR006015, Universal stress protein (Usp); IPR006016, UspA,domain-containing
	Sjc_0210600	IPR006015, Universal stress protein (Usp); IPR014729,Rossmann-like alpha/beta/alpha sandwich fold;IPR006016, UspA, domain-containing
	Sjc_0011870	ko:K06174 ATP-binding cassette, sub-family E, member 1, putative
	Sjc_0094860	ko:K09510 DnaJ homolog, subfamily B, member 4, putative
Muscular movement	Sjc_0044580	IPR002048, Calcium-binding EF-hand, domain-containing
	Sjc_0304180	ko:K10373 tropomyosin 1, putative
	Sjc_0211400	Myosin regulatory light chain A, smooth adductor muscle, putative
	Sjc_0102250	IPR002048, Calcium-binding EF-hand, domain-containing
	Sjc_0202230	ko:K09377 cysteine and glycine-rich protein, putative
	Sjc_0024050	Alpha-adducin, putative
	Sjc_0026420	Troponin I 4, putative
	Sjc_0106490	PDZ and LIM domain protein 3, putative
	Sjc_0012060	ko:K06094 lethal giant larvae, putative
Energy metabolism	Sjc_0214350	ko:K06942 OLA1; Obg-like ATPase 1, putative
	Sjc_0200590	ko:K00933 creatine kinase [EC2.7.3.2], putative
	Sjc_0049690	ATP synthase subunit O, mitochondrial precursor, putative
	Sjc_0000230	ko:K01509 adenosinetriphosphatase [EC3.6.1.3], putative
	Sjc_0205700	ko:K00382 dihydrolipoamide dehydrogenase [EC1.8.1.4], putative
Transport	Sjc_0304010	ko:K08495 golgi SNAP receptor complex member 1, putative
	Sjc_0209790	ko:K02150 V-type H+-transporting ATPase subunit E, putative
	Sjc_0035720	ko:K09500 T-complex protein 1 subunit theta, putative
	Sjc_0300180	Coatomer subunit gamma-2, putative
	Sjc_0071910	ko:K07936 GTP-binding nuclear protein Ran, putative
	Sjc_0005410	ko:K07881 Ras-related protein Rab-14, putative
	Sjc_0124170	Intersectin-1, putative
	Sjc_0207310	ko:K07949 ADP-ribosylation factor-like 5A, putative
	Sjc_0080950	AP-2 complex subunit alpha-2, putative
Lipid metabolism	Sjc_0213210	ko:K10258 enoyl reductase, putative
	Sjc_0027600	ko:K01648 ATP citrate (pro-S)-lyase [EC2.3.3.8], putative
	Sjc_0303020	ko:K01802 peptidylprolyl isomerase [EC5.2.1.8], putative
Cell proliferation	Sjc_0014710	ko:K02449 cyclin-dependent kinase 10, putative
	Sjc_0051340	High mobility group protein DSP1, putative
	Sjc_0300100	ko:K02605 origin recognition complex subunit 3, putative

Secondly, compared with 18 dpi males, more enzymes associated with glycometabolism, lipid metabolism, and energy metabolism (e.g., transketolase, Obg-like ATPase, and enoyl reductase) were acetylated in 28 dpi males. Maintaining pairing requires substantial energy. Therefore, the enrichment of acetylated metabolic enzymes might be associated with the increased metabolic activities in adult males, indicating that acetylation serves as a mean to modulate metabolic activities.

Thirdly, contrary to females, more proteins associated with cytoskeleton proteins were acetylated in 28 dpi males than in 18 dpi males. As previously mentioned, acetylation of actin and tubulin promotes muscular activity (Sadoul et al., 2011; Li and Yang, 2015). Therefore, acetylation of cytoskeleton proteins in 28 dpi males indicates an increased muscular activity, which is believed to be essential to maintain male-female pairing and their attachment to venous wall.

Finally, compared with 18 dpi males, more proteins associated with environmental stress resistance (e.g., universal stress protein) were acetylated in 28 dpi males. After pairing, only males are mainly exposed to the host blood. Both the free radicals from the oxygen rich environment and the host immune system could damage the parasites. We speculated that the acetylation of stress proteins might increase its stress resistance activity and thus protected worms from the toxicity of the free radicals and the attack of the host immune system.

### Collaborative impact of acetylation regulation and transcriptional regulation in *S. japonicum* development

Previously a transcriptomic study was carried out in our lab to describe the gene expression profiles from paring to sexual maturation in male and female schistosomes (Wang et al., 2017). It showed that in females, expression of genes associated with protein translation and macromolecules synthesis was up-regulated after 20 dpi, whereas expression of genes associated with muscular movement was down-regulated. In males, expression of genes associated with muscular development, movement regulation, signal transduction, and external signal perception were up-regulated after 20 dpi. Biologically, female motility indeed becomes less active after sexual maturation while adult males become more muscular and more active. Meanwhile, it is obvious that energy consumption and metabolic activities increased in adult worms. Therefore, we found that in many aspects, both the changes of the transcription profiles and the change of acetylation profiles seemed to be consistent with the physiological and morphological changes during schistosome development. As both the transcriptional regulation and post-translational acetylation has an impact on gene functional output, we propose that the change of a certain biological function at different developmental stages might be the collaborative result of transcriptional regulation and PTMs including acetylation.

Combining the transcriptomic study and acetylomic study, we hereby outlined an overall biological changes in schistosome sexual maturation: after pairing, females were protected by males and focused on egg laying, their muscular system and nerve system were less required and thus degenerated; as for males, to maintain pairing and dominate movement, their muscular activity and signal transduction activity were enhanced. Moreover, in adult worms, glycometabolism alone no longer provided sufficient energy and nutrition for egg laying (females) and maintaining the pairing (males). Therefore, lipid metabolism became more active to generate additional energy and nutrition.

### Mechanism of acetylation modulation in *S. japonicum*

Our study suggested acetylation participated in the modulation of a variety of biological functions during schistosome development through the following mechanism: firstly, by weakening the interaction between DNA and histone, and enhancing the binding of transcriptional factor and DNA, thus activating the transcription and translation of the target genes; secondly, by modulating glycometabolism and lipid metabolism, and hence maintaining the dynamic balance of energy supply during schistosome development; thirdly, by modulating muscular movement at different developmental stages; fourthly, by promoting proteins digestion and accelerating protein degradation.

## Conclusions

In summary, we presented the first comparative study of proteome-wide protein lysine acetylation in juvenile and adult *S. japonicum*. Our study indicated that by transcriptional regulation and PTMs including protein acetylation participated in the modulation of a variety of biological functions during schistosome development. Our study also deepened our understanding of the mechanism of *S. japonicum* development. As acetylation plays important regulatory roles in development, we believe that by targeting key proteins and disrupting their normal acetylation status, the sexual maturation of *S. japonicum*, which is essential for the egg production and the transmission of schistosomiasis, can be interrupted. Therefore, further studies should focus on analyzing the functions of these acetylated proteins to provide clues for the development of new anti-schistosome strategies.

## Author contributions

WH and XZ conceived and designed the study. NZ, ML, and LH performed the experiments. QL, NZ, HS, and WH analyzed the data. BX and XM prepared the worms. QL, NZ, and WH wrote the paper. All authors read and approved the final manuscript.

### Conflict of interest statement

The authors declare that the research was conducted in the absence of any commercial or financial relationships that could be construed as a potential conflict of interest.

## Supplementary material

Figure S1 Four representative MS results showing the acetylation. **(A)** H2A at K5 and K8; **(B)** H2B at K15 and K17; **(C)** H3 at K23; **(D)** H4 at K59. Each peptide was fragmented by MS/MS and the fragments observed were consistent with the sequence of the peptide as shown on top of each MS/MS spectrum. Note that b ions are counting from N-terminus and y ions from C-terminus.

Figure S2 GO terms enrichment (Biological Process, Molecular Function, Cellular Component) analysis of acetylated proteins in four samples. **(A)** 18 dpi female; **(B)** 18 dpi male; **(C)** 28 dpi female; **(D)** 28 dpi male. Red bars represent the reference, green bars represent acetylated proteins.

## Abbreviations

*S. japonicum*: *Schistosoma japonicum*
dpi: days post infection
PZQ: Praziquantel
PTM: post-translational modification
LC-ESI-MS/MS: liquid chromatography electrospray ionization tandem mass spectrometry
PBS: phosphate-buffered saline
DTT: dithiothreitol
FDR: false discovery rate
CID: collision-induced dissociation
GO: Gene ontology
KEGG: Kyoto Encyclopedia of Genes and Genomes
TCA cycle: tricarboxylic acid cycle.
