# Supplementary figures and images for: Comparative Analysis of Proteome-Wide Lysine Acetylation in Juvenile and Adult Schistosoma japonicum
Source: Front Microbiol. 2017 Nov 21;8:2248. doi: 10.3389/fmicb.2017.02248 (PMC5715381; doi:10.3389/fmicb.2017.02248)

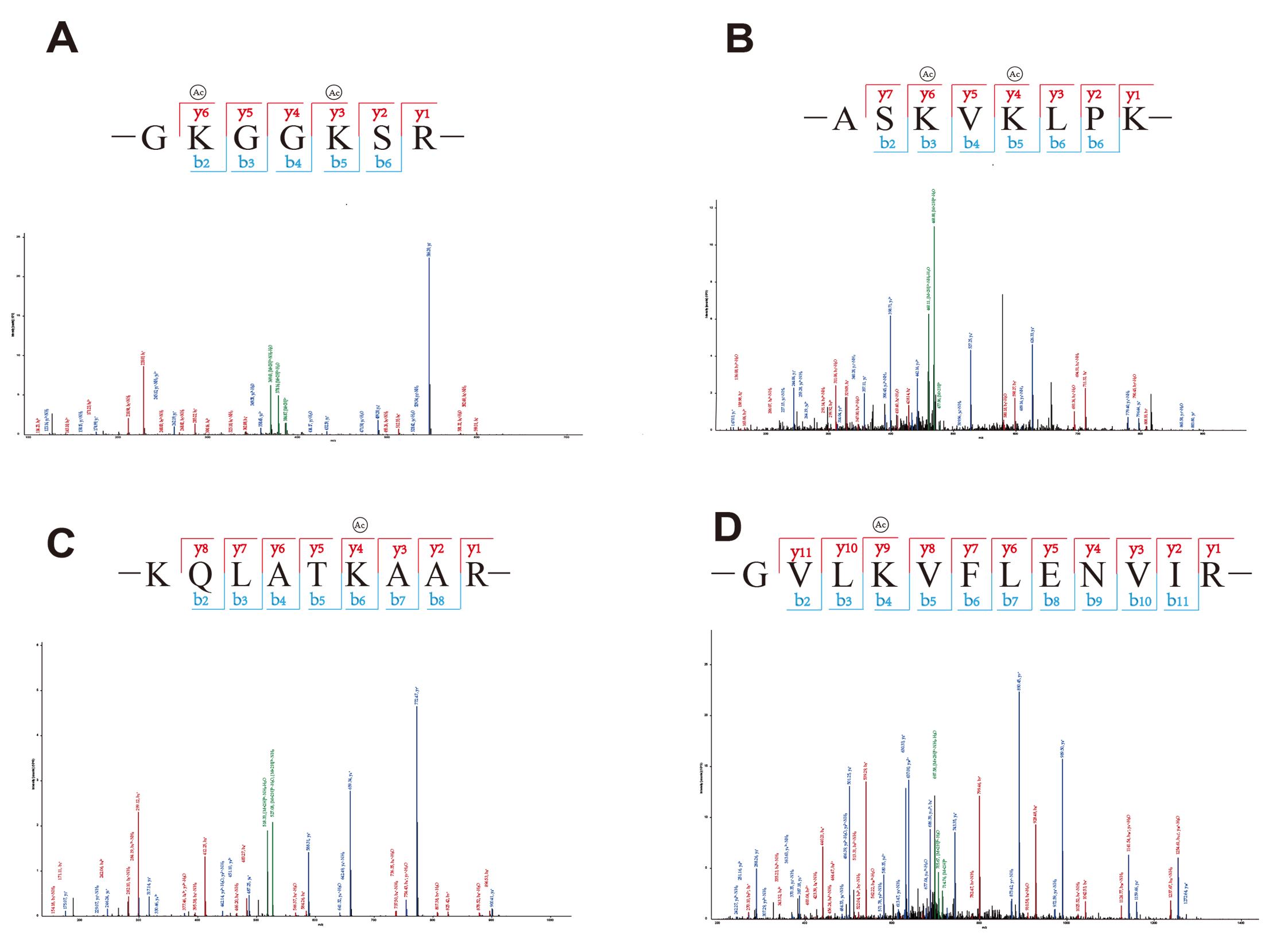

Supplement: Figure S1 — Four representative MS results showing the acetylation. (A) H2A at K5 and K8; (B) H2B at K15 and K17; (C) H3 at K23; (D) H4 at K59. Each peptide was fragmented by MS/MS and the fragments observed were consistent with the sequence of the peptide as shown on top of each MS/MS spectrum. Note that b ions are counting from N-terminus and y ions from C-terminus. [file Image1.JPEG]

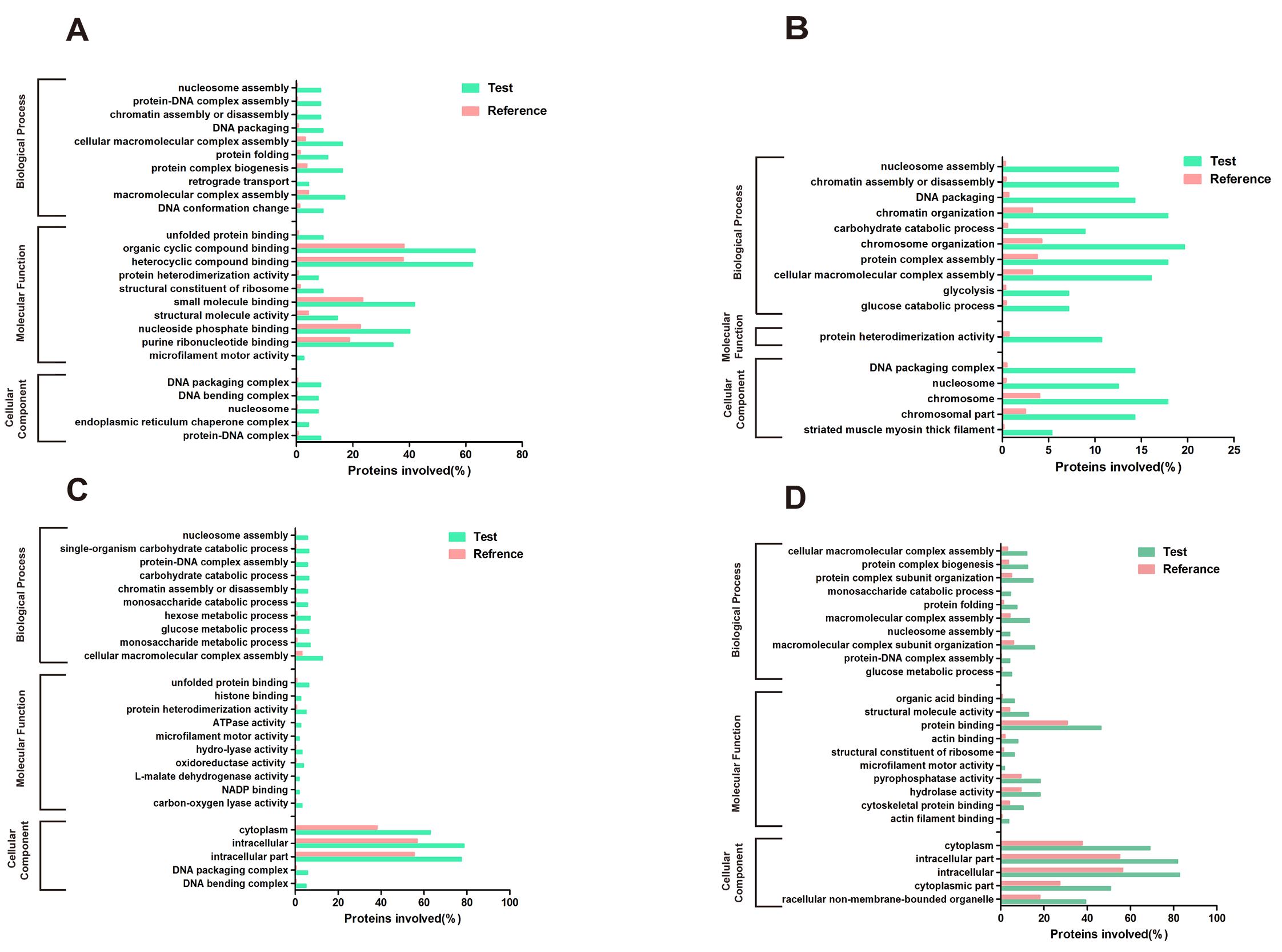

Supplement: Figure S2 — GO terms enrichment (Biological Process, Molecular Function, Cellular Component) analysis of acetylated proteins in four samples. (A) 18 dpi female; (B) 18 dpi male; (C) 28 dpi female; (D) 28 dpi male. Red bars represent the reference, green bars represent acetylated proteins. [file Image2.JPEG]
