# Supplementary material for: Comparative Analysis of Proteome-Wide Lysine Acetylation in Juvenile and Adult Schistosoma japonicum
Source: Front Microbiol. 2017 Nov 21;8:2248. doi: 10.3389/fmicb.2017.02248 (PMC5715381; doi:10.3389/fmicb.2017.02248)
Supplement: Supplementary file 4 [file Table2.DOCX]

**Supplementary Table S2** The shared proteins identified in all samples

| **Protein ID** | **Anotation** |
| --- | --- |
| **histone protein** |  |
| Sjc_0078660 | Histone H1-beta, late embryonic, putative |
| Sjc_0016080 | Histone H2B, gonadal, putative |
| Sjc_0058210 | Histone H2A.x, putative |
| Sjc_0016100 | Histone H4, putative |
| Sjc_0063110 | Histone H2A type 2-B, putative |
| **pre-mRNA processing** | |
| Sjc_0066380 | Heterogeneous nuclear ribonucleoprotein L, putative |
| Sjc_0111740 | Heterogeneous nuclear ribonucleoprotein K, putative |
| **transcription** |  |
| Sjc_0076820 | PCI domain-containing protein 2, putative |
| Sjc_0034190 | ko:K03138 transcription initiation factor TFIIF alpha subunit, putative |
| Sjc_0304610 | Endophilin-B1, putative |
| Sjc_0055670 | Probable small nuclear ribonucleoprotein Sm D2, putative |
| Sjc_0054280 | ko:K03231 elongation factor EF-1 alpha subunit, putative |
| Sjc_0200160 | ko:K09250 cellular nucleic acid-binding protein, putative |
| Sjc_0009730 | CREB-binding protein, putative |
| Sjc_0112250 | ko:K04498 E1A/CREB-binding protein, putative |
| **translation** |  |
| Sjc_0200460 | ko:K02927 large subunit ribosomal protein L40e, putative |
| Sjc_0011460 | ko:K02901 large subunit ribosomal protein L27e, putative |
| **Universal stress protein** | |
| Sjc_0061560 | IPR006015,Universal stress protein (Usp);IPR006016,UspA,domain-containing |
| Sjc_004468 | similar to Heat shock 70 kDa protein homolog, putative |
| **nuclear import protein** | |
| Sjc_0070960 | Importin subunit alpha-3, putative |
| **cytoskeletal protein and dynein proteins** | |
| Sjc_0027340 | Paramyosin, putative |
| **Metabolic enzymes** |  |
| Sjc_0025800 | ko:K00134 glyceraldehyde 3-phosphate dehydrogenase [EC1.2.1.12], putative |
| Sjc_0063550 | ko:K01830 prostaglandin-H2 D-isomerase [EC5.3.99.2], putative |
| Sjc_0304750 | ko:K01834 phosphoglycerate mutase [EC5.4.2.1], putative |
| Sjc_0303480 | ko:K01914 aspartate--ammonia ligase [EC6.3.1.1], putative |
| Sjc_0030210 | ko:K06630 tyrosine 3-monooxygenase/tryptophan 5-monooxygenase activation, putative |
| Sjc_0088510 | E3 SUMO-protein ligase RanBP2, putative |
| Sjc_0209670 | ko:K01689 enolase [EC4.2.1.11], putative |
| Sjc_0031630 | ko:K01623 fructose-bisphosphate aldolase, class I [EC4.1.2.13A], putative |
| Sjc_0212050 | ko:K00939 adenylate kinase [EC2.7.4.3], putative |
| Sjc_0120450 | ko:K00680 Mak3 homolog [EC:2.3.1.-], putative |
| Sjc_0031810 | ko:K00680 N-acetyltransferase ARD1 homolog [EC:2.3.1.-], putative |
| Sjc_0076300 | ko:K05024 chloride intracellular channel 4, putative |
| Sjc_0004470 | Epidermal retinal dehydrogenase 2, putative |
| **Other proteins** |  |
| Sjc_0007580 | ko:K06669 structural maintenance of chromosome 3 (chondroitin sulfate, putative |
| Sjc_0025560 | expressed protein |
| Sjc_0200380 | ko:K05862 voltage-dependent anion channel, putative |
| Sjc_0208800 | Receptor expression-enhancing protein 5, putative |
| Sjc_0047360 | Calcium-transporting ATPase sarcoplasmic/endoplasmic reticulum type, putative |
| Sjc_0217610 | ko:K10636 autocrine motility factor receptor, putative |
| Sjc_0050230 | IPR007648,Mitochondrial ATPase inhibitor, IATP,domain-containing |
| Sjc_0082530 | ko:K09493 T-complex protein 1 subunit alpha, putative |
