# Supplementary material for: Comparative Analysis of Proteome-Wide Lysine Acetylation in Juvenile and Adult Schistosoma japonicum
Source: Front Microbiol. 2017 Nov 21;8:2248. doi: 10.3389/fmicb.2017.02248 (PMC5715381; doi:10.3389/fmicb.2017.02248)
Supplement: Supplementary file 5 [file Table3.DOCX]

**Supplementary Table S3** Proteins with more than 4 acetylated sites

| **Proteins** | **Anotation** | **Acetylated sites** |
| --- | --- | --- |
| **18dpi-female** |  |  |
| Sjc_0063120 | Histone H3, putative | 6 |
| Sjc_0054280 | ko:K03231 elongation factor EF-1 alpha subunit, putative | 7 |
| Sjc_0016100 | Histone H4, putative | 8 |
| Sjc_0016080 | Histone H2B, gonadal, putative | 6 |
| **18dpi-male** |  |  |
| Sjc_0016080 | Histone H2B, gonadal, putative | 6 |
| **28dpi-female** |  |  |
| Sjc_0200380 | ko:K05862 voltage-dependent anion channel, putative | 5 |
| Sjc_0063550 | ko:K01830 prostaglandin-H2 D-isomerase [EC5.3.99.2], putative | 5 |
| Sjc_0063120 | Histone H3, putative | 5 |
| Sjc_0062310 | ko:K05863 solute carrier family 25 | 5 |
| Sjc_0054280 | elongation factor EF-1 alpha subunit, putative | 6 |
| Sjc_0044680 | similar to Heat shock 70 kDa protein homolog, putative | 6 |
| Sjc_0044660 | ko:K04079 molecular chaperone HtpG, putative | 5 |
| Sjc_0026230 | ko:K04043 molecular chaperone DnaK, putative | 5 |
| Sjc_0016100 | Histone H4, putative | 5 |
| Sjc_0016080 | Histone H2B, gonadal, putative | 7 |
| Sjc_0001030 | ko:K01802 peptidylprolyl isomerase [EC5.2.1.8], putative | 6 |
| Sjc_0054830 | ko:K10352 myosin heavy chain, putative | 4 |
| **28dpi-male** |  |  |
| Sjc_0211970 | ko:K00850 6-phosphofructokinase [EC2.7.1.11], putative | 5 |
| Sjc_0209670 | ko:K01689 enolase [EC4.2.1.11], putative | 10 |
| Sjc_0134610 | Myosin heavy chain, striated muscle, putative | 5 |
| Sjc_0112250 | ko:K04498 E1A/CREB-binding protein, putative | 5 |
| Sjc_0063550 | ko:K01830 prostaglandin-H2 D-isomerase [EC5.3.99.2], putative | 14 |
| Sjc_0063120 | Histone H3, putative | 5 |
| Sjc_0054830 | ko:K10352 myosin heavy chain, putative | 27 |
| Sjc_0054280 | ko:K03231 elongation factor EF-1 alpha subunit, putative | 10 |
| Sjc_0044680 | similar to Heat shock 70 kDa protein homolog, putative | 7 |
| Sjc_0044660 | ko:K04079 molecular chaperone HtpG, putative | 7 |
| Sjc_0038300 | ko:K00799 glutathione S-transferase [EC2.5.1.18], putative | 11 |
| Sjc_0031630 | ko:K01623 fructose-bisphosphate aldolase, class I [EC4.1.2.13A], putative | 7 |
| Sjc_0027340 | Paramyosin, putative | 7 |
| Sjc_0016100 | Histone H4, putative | 9 |
| Sjc_0016080 | Histone H2B, gonadal, putative | 7 |
| Sjc_0009730 | CREB-binding protein, putative | 7 |
| Sjc_0001030 | ko:K01802 peptidylprolyl isomerase [EC5.2.1.8], putative | 6 |
